# FOXO Regulates Neuromuscular Junction Homeostasis During *Drosophila* Aging

**DOI:** 10.3389/fnagi.2020.567861

**Published:** 2021-01-27

**Authors:** Allison Birnbaum, Maggie Sodders, Mark Bouska, Kai Chang, Ping Kang, Elizabeth McNeill, Hua Bai

**Affiliations:** ^1^Department of Genetics, Development, and Cell Biology, Iowa State University, Ames, IA, United States; ^2^Department of Cell, Developmental and Integrative Biology, University of Alabama Birmingham, Birmingham, AL, United States; ^3^Department of Food Science and Human Nutrition, Iowa State University, Ames, IA, United States

**Keywords:** FOXO, NMJ, MAPK, p38, Rab7, late endosome, aging

## Abstract

The transcription factor foxo is a known regulator of lifespan extension and tissue homeostasis. It has been linked to the maintenance of neuronal processes across many species and has been shown to promote youthful characteristics by regulating cytoskeletal flexibility and synaptic plasticity at the neuromuscular junction (NMJ). However, the role of foxo in aging neuromuscular junction function has yet to be determined. We profiled adult Drosophila foxo- null mutant abdominal ventral longitudinal muscles and found that young mutants exhibited morphological profiles similar to those of aged wild-type flies, such as larger bouton areas and shorter terminal branches. We also observed changes to the axonal cytoskeleton and an accumulation of late endosomes in foxo null mutants and motor neuron-specific foxo knockdown flies, similar to those of aged wild-types. Motor neuron-specific overexpression of foxo can delay age-dependent changes to NMJ morphology, suggesting foxo is responsible for maintaining NMJ integrity during aging. Through genetic screening, we identify several downstream factors mediated through foxo-regulated NMJ homeostasis, including genes involved in the MAPK pathway. Interestingly, the phosphorylation of p38 was increased in the motor neuron-specific foxo knockdown flies, suggesting foxo acts as a suppressor of p38/MAPK activation. Our work reveals that foxo is a key regulator for NMJ homeostasis, and it may maintain NMJ integrity by repressing MAPK signaling.

## Introduction

Aging involves the progressive functional decline of cellular mechanisms and tissue integrity (Rose, 1994; Partridge and Barton, 1996; Lopez-Otin et al., 2013). In the adult, this results in a gradual decline of synaptic contacts to skeletal muscle tissue, resulting in a loss of strength and muscle mass (Hall and Sanes, 1993). The neuromuscular junction (NMJ) serves as the synaptic interface between the branched terminals of motor neurons and the skeletal muscle fibers (Punga and Ruegg, 2012). The NMJ is highly dynamic in response to cellular signals and stressors, and a dysregulation of molecular processes in both the pre- and post-synaptic regions can lead to the onset of neurodegenerative diseases (Gonzalez-Freire et al., 2014; Monani and De Vivo, 2014). With aging there is impairment of regulatory systems such as autophagy and redox homeostasis at the NMJ, which can lead to reactive oxygen species (ROS) accumulation followed by organelle damage and cell death (Li et al., 2018; Stefanatos and Sanz, 2018). Synaptic plasticity, which allows for the maintenance of functional activity to protect against degeneration, can decline during aging, resulting in decreased neuronal responsiveness and synaptic deterioration (Bergado and Almaguer, 2002; Kempsell and Fieber, 2015; Wagner et al., 2015).

The *Drosophila* NMJ synapse utilizes glutamate as the primary neurotransmitter and has postsynaptic densities that behave similarly to the AMPA-type receptors in the vertebrate central nervous system (Menon et al., 2013). The Drosophila neuromuscular junction system has been well-stereotyped in larvae, but has only more recently begun to be characterized in the adult fly (Rivlin et al., 2004; Hebbar et al., 2006; Beramendi et al., 2007; Wagner et al., 2015; Lopez-Arias et al., 2017). These studies have also discovered a number of morphological changes that occur at the NMJ under normal aging conditions and can aid in understanding the mechanisms involved in age-dependent neuronal functional decline.

The foxo transcription factor family plays an important role in metabolism and development as well as stress resistance and lifespan (Accili and Arden, 2004; Greer and Brunet, 2005). foxo has long been characterized as a “longevity gene” and as a modulator of protein homeostasis (Kenyon et al., 1993; Martins et al., 2016). FOXO proteins can also function as regulators of neuronal homeostasis (Paik et al., 2009; Kim and Webb, 2017; Schaffner et al., 2018). FOXO-mediated pathways permit neuronal plasticity, which have downstream implications on cellular behavior and neuronal morphology (McLaughlin and Broihier, 2018). *Drosophila* FOXO (dFOXO, hereafter foxo) promotes cytoskeletal dynamics at the neuromuscular junction during larval development, where it responds to stimulation to allow rapid structural reorganization. In the Drosophila larvae NMJ, loss of foxo results in increased stability of microtubules through the upregulation of kinesin motor protein regulator Paverotti that also controls axon growth (Nechipurenko and Broihier, 2012; McLaughlin et al., 2016). In mammals, certain foxo isoforms show transcriptional regulation of cytoskeletal genes during development (de la Torre-Ubieta et al., 2010). FOXOs are also implicated in regulating autophagy in neurons under Parkinson's disease models (Xu et al., 2011; Pino et al., 2014; Schaffner et al., 2018). It has also been shown that aging results in the activation of pro-inflammatory signals and proteotoxic stress in mice brains, and the depletion of neuronal FOXOs causes premature occurrences of these events and induces motor function impairment (Hwang et al., 2018). Additionally, loss of foxo impacts synaptic function by reducing neurotransmitter release in both larval and adult drosophila neuromuscular junctions (Howlett et al., 2008; Mahoney et al., 2016). Aged synapses also show a reduction in evoked response and neurotransmission (Segal, 1982; Porras and Mora, 1995; Liu et al., 2013). These studied indicate that foxo is an important regulator of motor neuron function, and that foxo impacts the functional and structural integrity of the neuromuscular junction.

Our current knowledge of foxo supports the hypothesis that foxo activity promotes youthful NMJ morphology and provides necessary regulation of pathways involved in maintaining synaptic integrity and plasticity. However, how foxo impacts functional regulation of the NMJ during aging remains to be elucidated. Here, we show that loss of foxo causes morphological alterations to the NMJ in young adult flies and shares phenotypic characteristics with those of aging control organisms. We find that knocking down foxo specifically in the motor neuron results in changes to microtubule morphology and an upregulation of late endocytic vesicles in the nervous system. These events are also observed in middle-aged flies, indicating loss of foxo may lead to a disruption of motor neuron homeostasis. Overexpression of foxo in the motor neuron delays these age-related changes to NMJ morphology. Additionally, we identified potential pathways downstream of foxo that may promote aging and decline in synaptic function in the adult motor neurons. These findings provide insight into the role foxo plays in maintaining morphological and synaptic plasticity at the aging neuromuscular junction.

## Results

### Loss of Foxo Causes Morphological Changes to Bouton Size and Branch Length That Correspond to Aging

**Figure 1:**
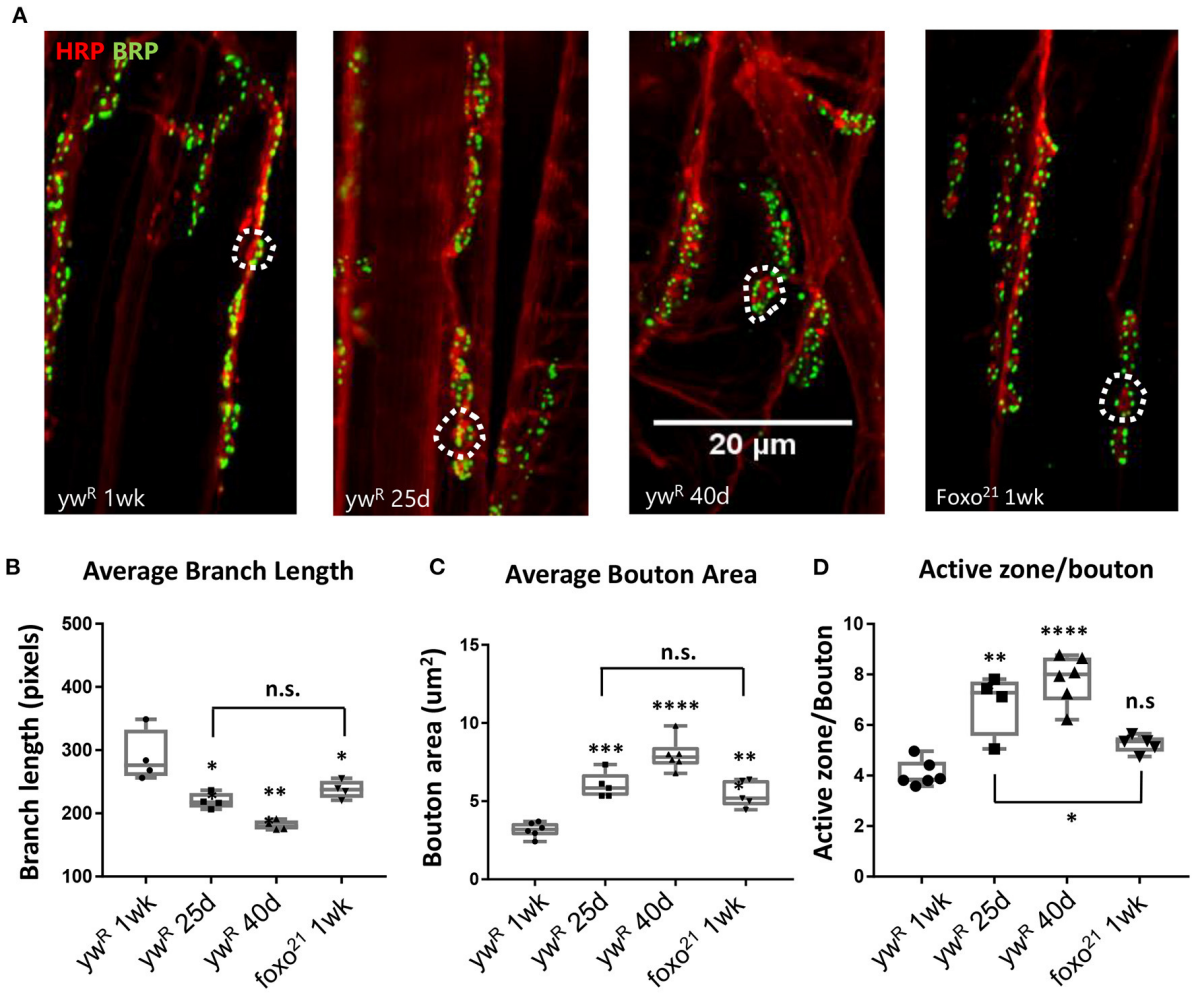
Loss of FOXO results in middle-aged NMJ morphology in the abdominal ventral muscle. **(A)** Representative images of the Abdominal VLM A3 segment for axon terminals with indicated genotypes and ages colabeled with Anti-BRP and anti-HRP. Bar 20 μM. **(B)** Average branch length. *P < 0.05, **P < 0.01. Bouton area for 1-week *foxo* flies is significantly larger than 1-week controls, but is comparable to 25-day control flies. **P < 0.01. **(C)** Average bouton area **P < 0.01, ***P > 0.001, ****P < 0.0001. **(D)** Quantification of changes in active zone number per bouton. *P < 0.05, **P < 0.01, ***P < 0.001. ywR 1 wk *n* = 6; 25 d *n* = 5; 40 d *n* = 6; foxo^21^ 1 wk *n* = 5. Number of boutons quantified per sample analyzed: ywR 1 wk *n* = 177, 25 d *n* = 75, 40 d *n* = 96, foxo^21^ 1 wk *n* = 100. Number of branches analyzed: ywR 1 wk *n* = 39, 25 d *n* = 39, 40 d *n* = 40, foxo^21^ 1 wk *n* = 39.

Normal aging causes alterations to the morphology of boutons and branches at the adult Drosophila neuromuscular junction (Beramendi et al., 2007; Wagner et al., 2015; Lopez-Arias et al., 2017). Based on previous literature, we selected three time points post eclosion, 6, 25, and 40 days to represent young, middle aged, and old adult flies. We visualized the A3 abdominal ventral longitudinal muscle (VLM) segment using immunofluorescence staining in female flies. We stained flayed adult abdominal pelts with a monoclonal antibody against bruchpilot (BRP) to mark active zones (AZs) and an antibody against horseradish peroxidase (HRP) to visualize neuronal tissue (Figure 1A). To obtain overall bouton number, we used an antibody against Disc-large (Dlg) to mark post-synaptic regions. Using the two NMJ's present at each A3, we quantified the six metrics characterized by Wagner et al. (2015) and found a significant difference between ages for several metrics, as expected. A significant decrease in branch length was observed with aging, along with a decrease in bouton number (Figure 1B, Table 1). We also found an expansion of bouton area with age, and this corresponded to an increase in number of AZs per bouton (Figures 1C,D, Table 1). However, the number of AZs observed in a 100 um^2^ region did not change between ages, which has been shown to remain constant during aging (Table 1, Wagner et al., 2015).

**Table 1:** Quantification of NMJ morphological characteristics of aging.

	**Size of boutons (um^**2**^)**	**Branch length (pixels)**	**AZs/bouton**	**AZs/synaptic area (100 um^**3**^)**	**Bouton number**	**Branch number**
ywR 1 wk	3.16 ± 1.84	290.6 ± 109.6	4.07 ± 0.51	24.14 ± 6.61	90.5 ± 18.41	18.25 ± 1.26
yw^R^ 25 d	6.10 ± 2.79	219.5 ± 55.28	6.86 ± 1.24	23.06 ± 8.22	71.6 ± 18.56	14.5 ± 0.71
yw^R^ 40 d	7.91 ± 4.23	180.7 ± 48.1	7.81 ± 0.95	21.09 ± 4.38	52.0 ± 11.86	15.75 ± 1.71
foxo^21^ 1 wk	5.40 ± 3.08	238.2 ± 69.66	5.25 ± 0.33	21.54 ± 3.825	111.5 ± 17.92	28.33 ± 7.57

*ywR is the control line for foxo21. Average bouton number: 1-week ywR v 1-week foxo21 (n.s.). 1-week ywR v 25-day ywR (n.s.). 1-week ywR v 40-day ywR (P < 0.05). 25-day ywR v 1-week foxo21 (P < 0.05). Branch number: 1-week ywR v 1-week foxo21 (P < 0.05). 1-week ywR v 25-day ywR (n.s.). 1-week ywR v 40-day ywR (n.s.). Bouton number and branch number represent both NMJs present at the A3 abdominal segment*.

We next examined how the loss of foxo effects NMJ morphology in the adult. We used the hypomorphic allele *foxo*^21^ and dissected the VLM of 1-week post eclosion animals and characterized the A3 muscle as described previously (Figure 1A). We found that *foxo* mutants had significantly larger bouton areas and shorter branches than their 1-week wild-type counterparts. However, the bouton area and branch length of *foxo* mutants was not significantly different from those seen in middle age control flies (Figures 1B,C, Table 1). The average active zone number between young wild-type and *foxo* mutants was not significantly different, but the *foxo*-null mutants had more average AZs per bouton compared to controls (Figure 1D, Table 1, Supplementary Figure 1). When evaluating the raw total number of active zones per bouton, a significant difference was detected between 1-week controls and *foxo* mutants, but not between 25-day controls and 1-week *foxo* mutants (Supplementary Figure 1). Additionally, we did not detect a significant difference in overall AZs per synaptic area in *foxo* mutants when compared to the three measured control age groups. We did find that *foxo* mutants on average had more branches and a higher bouton number than wild-type flies (Table 1), which may be a compensatory response to reduced neurotransmission under excitatory conditions seen in *foxo* mutants (Howlett et al., 2008; Mahoney et al., 2016; McLaughlin and Broihier, 2018). We also examined bouton size and branch length in 1-week old flies containing a null *foxo*^*C*431^ allele generated through CRISPR-Cas9 (Birnbaum et al., 2019). We found these null mutants also had enlarged boutons and shorter branch lengths compared to controls at a young age (Supplementary Figure 1). We validated that foxo protein levels were indeed reduced in our mutants (Supplementary Figure 1). Taken together, these results suggest that loss of foxo promotes aging-like morphological changes in boutons and synaptic branches at the adult neuromuscular junction.

### Loss of Foxo Results in Altered Cytoskeleton Structure in the Adult Neuromuscular Junction

**Figure 2:**
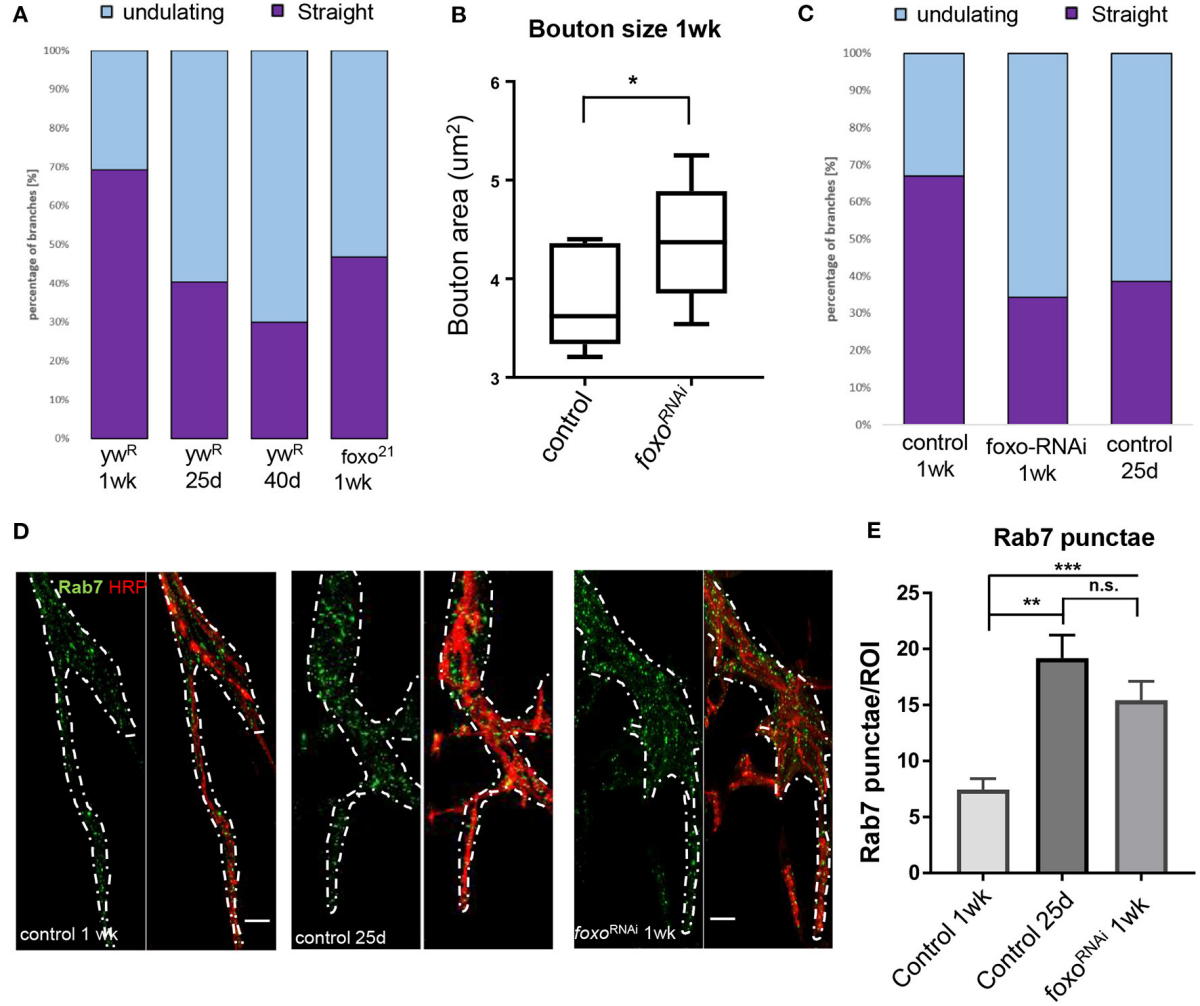
Aging and loss of FOXO cause changes in microtubule structure and endocytic activity. **(A)** Quantification of anti-Ac-Tub at A3 VLM at control aging timepoints and in *foxo* mutant. The mean fraction of straight branches was significantly reduced from the young control for all other columns (*P* < 0.05). **(B)** Average bouton area for ok6-GAL4>yw^R^ compared to ok6-GAL4>foxo-RNAi #1 (*P < 0.05). **(C)** Quantification of anti-Ac-Tub at A3 VLM at specified genotypes and ages. The % of straight branches of all other columns was significantly reduced from the young control (*P* < 0.05). **(D)** Representative images of main axon branchpoint with indicated genotypes and ages. Colabeled with Anti-Rab7 and anti-HRP. Nervous system tissue is outlined in white. Surrounding muscle tissue has been removed from the image. Bar 10 μM. **(E)** Number of Rab7 punctae detected per ROI in A3 segment. **P < 0.01, ***P < 0.001, n.s.- not significant. Number of animals analyzed for Ac-Tub: ywR 1 wk *n* = 3, 25 d *n* = 3, 40 d *n* = 3, foxo^21^ 1 wk *n* = 3, ok6-GAL4>yw^R^ 1 wk = 5, ok6-GAL4>yw^R^ 25 d = 5, ok6-GAL4> foxo-RNAi #1 1 wk = 4. Number of branches analyzed for Ac-Tub; ywR 1 wk *n* = 38, 25 d *n* = 37, 40 d *n* = 26, foxo^21^ 1 wk *n* = 46, ok6-GAL4>yw^R^ 1 wk = 70, ok6-GAL4>yw^R^ 25 d = 90, ok6-GAL4> foxo-RNAi #1 1 wk = 75. Number of animals analyzed for Rab7 quantification; ok6-GAL4>yw^R^ 1 wk = 7, ok6-GAL4>yw^R^ 25 d = 6, ok6-GAL4> foxo-RNAi #1 1 wk = 8.

Axonal degeneration is a common consequence of aging and arises from a number of alterations to cellular and molecular pathways (Salvadores et al., 2017). This can result in a withdrawal of axons from their synaptic sites, caused by changes in the underlying cytoskeleton (He et al., 2002; Manini et al., 2013). Foxo proteins are known to regulate motor neuron microtubule dynamics during development across many species, and can cause a decrease in microtubule stability, allowing for increased plasticity (de la Torre-Ubieta et al., 2010; Nechipurenko and Broihier, 2012; McLaughlin et al., 2016). However, how foxo effects microtubule dynamics in a post-developmental model has not been examined. We used an antibody against acetylated alpha tubulin (Ac-tub) which marks stable microtubules at our three aging time points. We constructed a 3D model of each A3 segment and assigned all branches containing acetylated a-tubulin one of two characteristics; straight, or undulating. We quantified the percentage of each tubulin morphology found the undulating morphology significantly increases between 1-week and 25 days, and between 25 and 40 days (Figure 2A, Supplementary Figure 2). In examining the wild-type flies, we found at the young time point around 70% of branches had a straight tubulin structure. As wild-type flies aged, we saw an increase in sinusoidal bends persisting throughout the branches, creating an undulating pattern (Supplementary Figure 2). This morphology can be associated with axonal retraction (He et al., 2002). These results show that there are changes that occur to the axonal microtubule structure with aging.

We next examined *foxo* mutants for any disruptions to the axonal microtubule morphology. *Foxo* mutants exhibited an increased number of undulating microtubules within branches and had significantly fewer straight microtubule bundles than the control at 1 week post eclosion (Supplementary Figure 2). When the 1-week *foxo* mutants were compared to the 25-day controls, there was no significant difference detected between the percentage of both undulating and straight microtubule branches (Figure 2A). To examine whether the change in microtubule morphology results from motor neuron specific foxo activity, we used a motor neuron specific *Ok6-Gal4* to knock down *foxo* specifically in the motor neuron. We quantified bouton size between control and foxo-knockdown flies and found that knock-down of *foxo* in the motor neuron induces an increase in bouton size (Figure 2B). We stained A3 VLMs against Ac-tub, and at 1-week post eclosion, and observed nearly a 2-fold decrease in the number of straight branches in knockdown flies compared to controls (*Ok6-Gal4*>*ywR)* (Figure 2C, Supplementary Figure 2). We also compared our 1-week *foxo* knockdown to 25-day controls and found there to be no significant difference between the percentage of undulating and straight microtubules within axonal branches (Figure 2C, Supplementary Figure 2). Thus, these findings suggest that foxo regulates bouton size and axonal microtubule morphology autonomously.

### Loss of Foxo Causes Alteration to the Endocytic Pathway in the Adult Neuromuscular Junction

Aging has been shown have disrupted endosome-lysosome trafficking and increased numbers of early endosomes and multivesicular bodies (prerequisites for late endosomes) at the synapse, which can result in neurodegeneration (Boaro et al., 1998; Nixon et al., 2008; Wagner et al., 2015; Colacurcio and Nixon, 2016). In *C. elegans*, late endosome formation is suppressed in organisms with delayed aging (Richardson et al., 2019). Additionally, late endosomes undergo retrograde transport to degrade cargo, and this transport declines with age (Milde et al., 2015; De Vos and Hafezparast, 2017). To observe changes in the endocytic degradation pathway, we used an antibody against Rab7 to mark late endosomes. We performed immunofluorescent staining at the A3 VLM on 1-week and 25-day adults. To our surprise, we observed Rab7-marked punctae around the axon branchpoint site and noticed a visible increase at this site in middle-aged flies. To quantify the number of Rab7 vesicles, we generated circular regions of interest (ROIs) of the same size and used HRP to identify z-slices containing the axon. The slice with the highest punctae number for each ROI was collected and averaged for each specimen. We found the 25-day adult flies had more than 2-fold more Rab7 punctae per ROI associated with the axon branch than the 1-week adults, demonstrating there is an increase in the number of late endosomes during aging (Figures 2D,E).

We next examined the effects of motor neuron specific knockdown of *foxo* in late endosome signaling. We found that *foxo* KD flies had significantly more punctae per ROI than the young controls in nervous system tissue (Figures 2D,E, Supplementary Figure 4). We tested a second foxo-RNAi line (foxo-RNAi #2) and again observed significantly more Rab7 punctae at the branchpoint than controls (Supplementary Figure 3). To test whether the morphological changes of foxo knockdown flies are due to the developmental defects, we examined for Rab7 accumulation, bouton size and ac-tubulin structure from the female flies 2 day poste eclosion. We found no significant differences were detected between control and *foxo*-RNAi flies (Supplementary Figure 4). Overall, both aging and *foxo* knockdown result in an increase in the number of late endosomes around the branchpoint of motor neuron, suggesting a potential role of foxo in age-related alterations in endocytic pathway.

### Overexpression of *foxo* Rescues NMJ Aging Phenotypes

**Figure 3:**
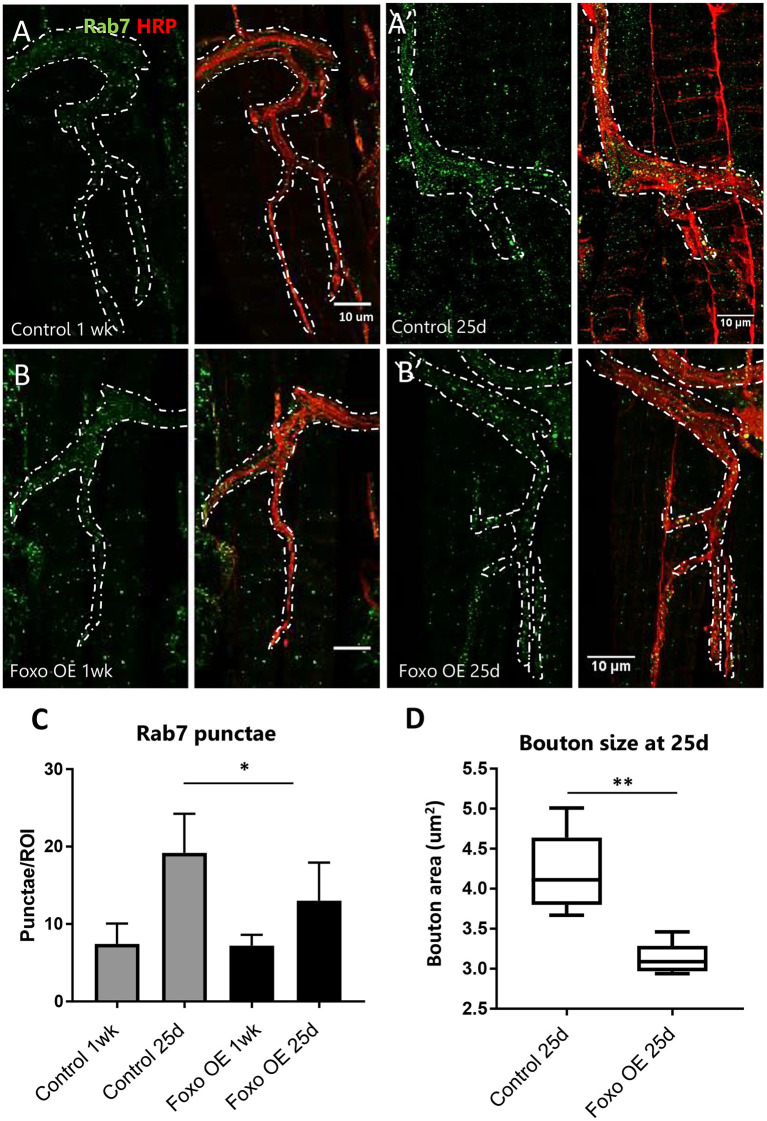
Overexpression of motor neuron FOXO delays aging phenotypes **(A,B)**. Representative figures of control and FOXO-overexpression at 1-week, **(A',B')** and 25 days post eclosion. Colabelling with anti-Rab7 and anti-HRP. Scale bar 10 μm. **(C)** Rab7 quantification for A3 ROIs for indicated genotypes and ages *P < 0.05. **(D)** Average bouton area for 25-day old FOXO overexpression and control **P < 0.01. Number of animals analyzed for Rab7 quantification; ok6-GAL4>yw^R^ 1 wk = 7, ok6-GAL4>yw^R^ 25 d = 6, ok6-GAL4> UAS-foxo 1 wk = 4. ok6-GAL4> UAS-foxo 25 d = 7. Number of animals analyzed for bouton size: ok6-GAL4>yw^R^ 25 d = 5, ok6-GAL4> UAS-foxo 25 d = 5.

To test if overexpression of *foxo* can restore age-related NMJ defects, we crossed *Ok6-GAL4* to *UAS-foxo* flies to express the wild-type *foxo* transgene specifically in the motor neuron. At 1-week post eclosion, no differences were detected in Rab7 number between controls and *foxo* overexpression flies (Figures 3A–C). At 25 days, *foxo* overexpression flies exhibited significantly fewer late endosomes than control flies (Figures 3A',B',C). Consistently, *foxo* overexpression attenuated age-related increases in bouton size (Figure 3D). To test for the effectiveness of foxo overexpression, we measured the expression of a known foxo target gene, Thor (4EBP) (Junger et al., 2003). We found a 2-fold increase in Thor expression in *foxo* overexpression flies (Supplementary Figure 3). Taken together, these results indicate that overexpression of *foxo* can preserve motor neuron morphology and endocytic activities.

### MAPK and Activin Signaling Genetically Interact With Foxo to Control NMJ Homeostasis

**Figure 4:**
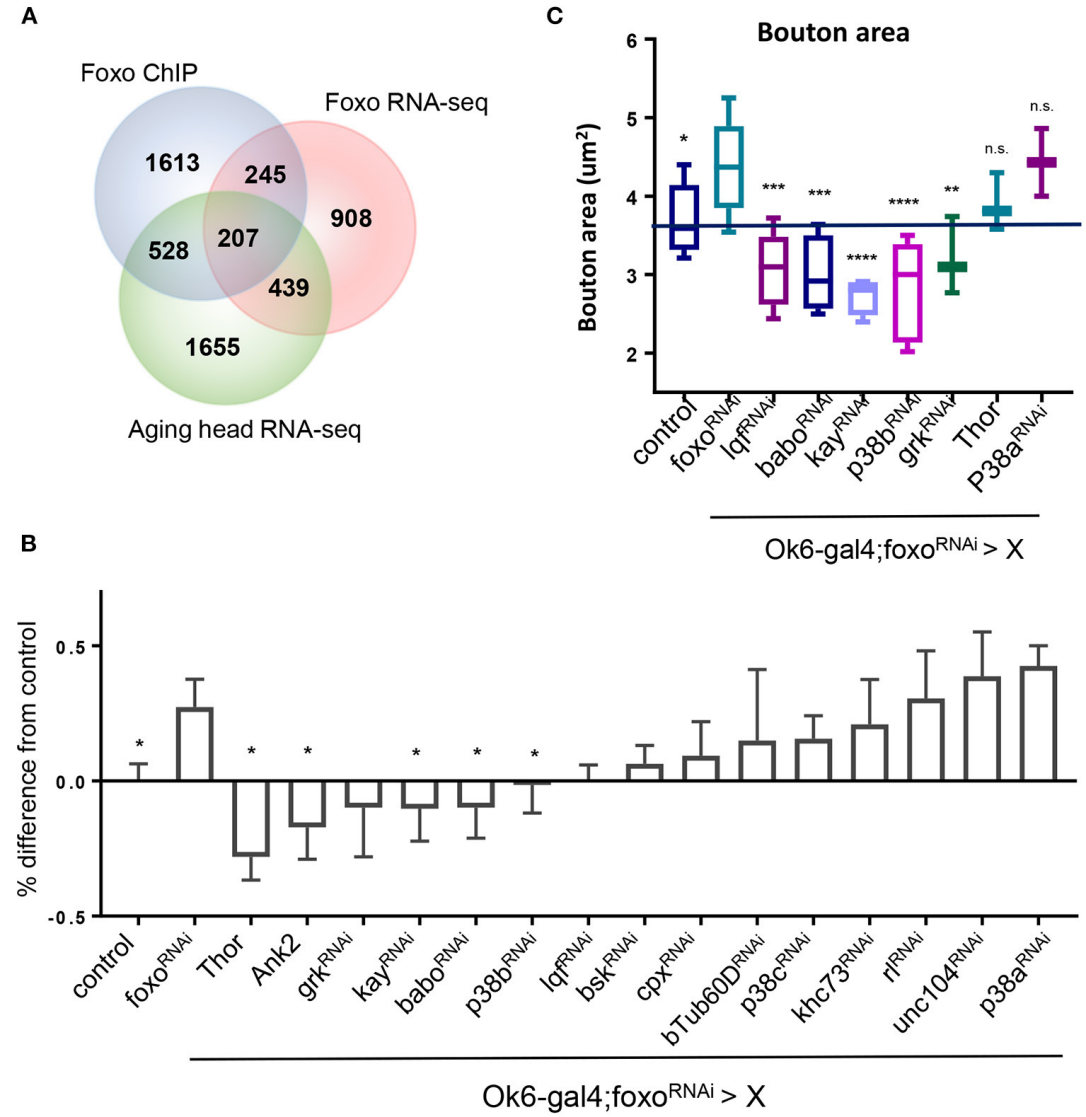
MAPK acts downstream of FOXO to regulate NMJ pathways. **(A)** Venn diagram overlap of ChIP-seq, head foxo^C431^ RNA-seq, and aged head RNA-seq: For RNA-seq data {−0.15 > log2FC > 0.15, *P* < 0.05}. {FOXO ChIP FC >1.5, FDR < 0.05}. **(B)** Quantification of Rab7 for double mutant flies. Values are set as a percentage with ok6>yw^R^ as baseline. All significant values are compared to foxo^RNAi^ (in ok6-GAL4 background) *P < 0.05. **(C)** Average bouton size quantification for indicated genotypes. Control in Ok6-GAL4 background. *P < 0.05, **P < 0.01, ***P < 0.001, ****P < 0.0001, n.s., not significant. Number of animals used for **(B)** in order from left to right: *n* = 22; 13; 7; 4; 3; 6; 10; 7; 4; 6; 5; 4; 6; 6; 6; 3; 3. Number of animals used for **(C)** bouton size in order from left to right: *n* = 14; 11; 4; 5; 4; 5; 3; 3; 2. Number of animals used for **(B)** c-Tub: ok6-GAL4; foxo-RNAi >ywR = 4, ok6-GAL4; foxo-RNAi> babo-RNAi = 6, ok6-GAL4; foxo-RNAi > p38b-RNAi = 5. Number of branches used for **(B)** c-Tub: ok6-GAL4; foxo-RNAi >ywR = 75, ok6-GAL4; foxo-RNAi> babo-RNAi = 93, ok6-GAL4; foxo-RNAi > p38b-RNAi = 80.

In order to dissect the mechanisms underlying foxo-regulated NMJ homeostasis, we conducted a genetic screening to uncover the downstream targets of foxo in the motor neuron. The candidate genes were selected from our previous foxo ChIP-Seq and transcriptome analyses (Birnbaum et al., 2019). We selected 207 candidate genes with positive foxo binding (fold change >1.5) and differential expression (fold change>1.25, *p* < 0.05) (Figure 4A, Supplementary Table). Interestingly, these candidate genes were enriched for biological processes involving neurotransmitter secretion, cytoskeletal organization, and signaling pathways such as MAPK (Supplementary Table).

Among all identified candidate genes, we prioritized 15 candidates in the genetic screening. Rab7 and bouton morphology were used as NMJ readouts (Table 2). We first generated a fly line by combining *Ok6-gal4* driver and *UAS-foxo-RNAi*. Through the screening, we identified four genes whose knockdown attenuated the accumulation of late endosome number in *foxo* KD flies. We also found that overexpression of two genes Thor and Ank2 significantly reduced Rab7 number of the *foxo* KD flies. Among identified target genes, three genes, JNK transcription factor *kay* (*Kayak*), type I activin receptor *babo* (*baboon*), and p38b MAP kinase (p38b), are known to have regulatory effects on axonal transport (Ellis et al., 2010; Drerup and Nechiporuk, 2013; Gibbs et al., 2018). Similarly, knockdown of five candidate genes (lqf, babo, kay, p38b, grk) blocked the induction of bouton size in *foxo* KD flies (Figure 4C). Thus, our findings indicate that TGF-β and MAPK signaling are the downstream effectors that mediate foxo-regulated NMJ homeostasis.

**Table 2:** Downstream candidate genes from FOXO ChIP-seq and RNA-seq.

**Gene symbol**	**Gene name**	**Function**	**ChIP FC**	**FOXO RNA-seq log2FC**	**Aging head RNA-seq log2FC**
rl	Rolled	ERK	4.73	0.343	0.159
Lqf	Liquid facets	Epsin1	2.67	0.386	1.132
Unc-104	Uncoordinated-104	Kinesin/KIF1A	2.33	−0.388	0.327
Khc-73	Kinesin heavy chain 73	Kinesin	1.94	−0.23	0.106
Ank2	Ankyrin 2	Microtubule associated protein	3.48	−0.433	0.468
Cpx	Complexin	SNARE regulator	3.68	−0.22	1.001
bTub60D	Beta–Tubulin at 60D	Microtubule building block	1.91	1.442	−1.147
Babo	Baboon	Activin receptor	3.71	0.244	0.258
Grk	Gurken	ERK ligand	2.61	0.292	−0.560
Thor	Thor	4EBP	3.24	−0.225	−0.137
Bsk	Basket	JNK	–	−0.343	0.529
Kay	Kayak	JNK Transcription factor	2.55	−0.059	−0.302
P38a	p38a MAP kinase	P38 kinase	–	0.211	−0.737
P38b	p38b MAP kinase	P38 kinase	–	0.133	0.358
p38c	p38c MAP kinase	P38 kinase	–	−0.305	−0.946

*FC, Fold change*.

**Figure 5:**
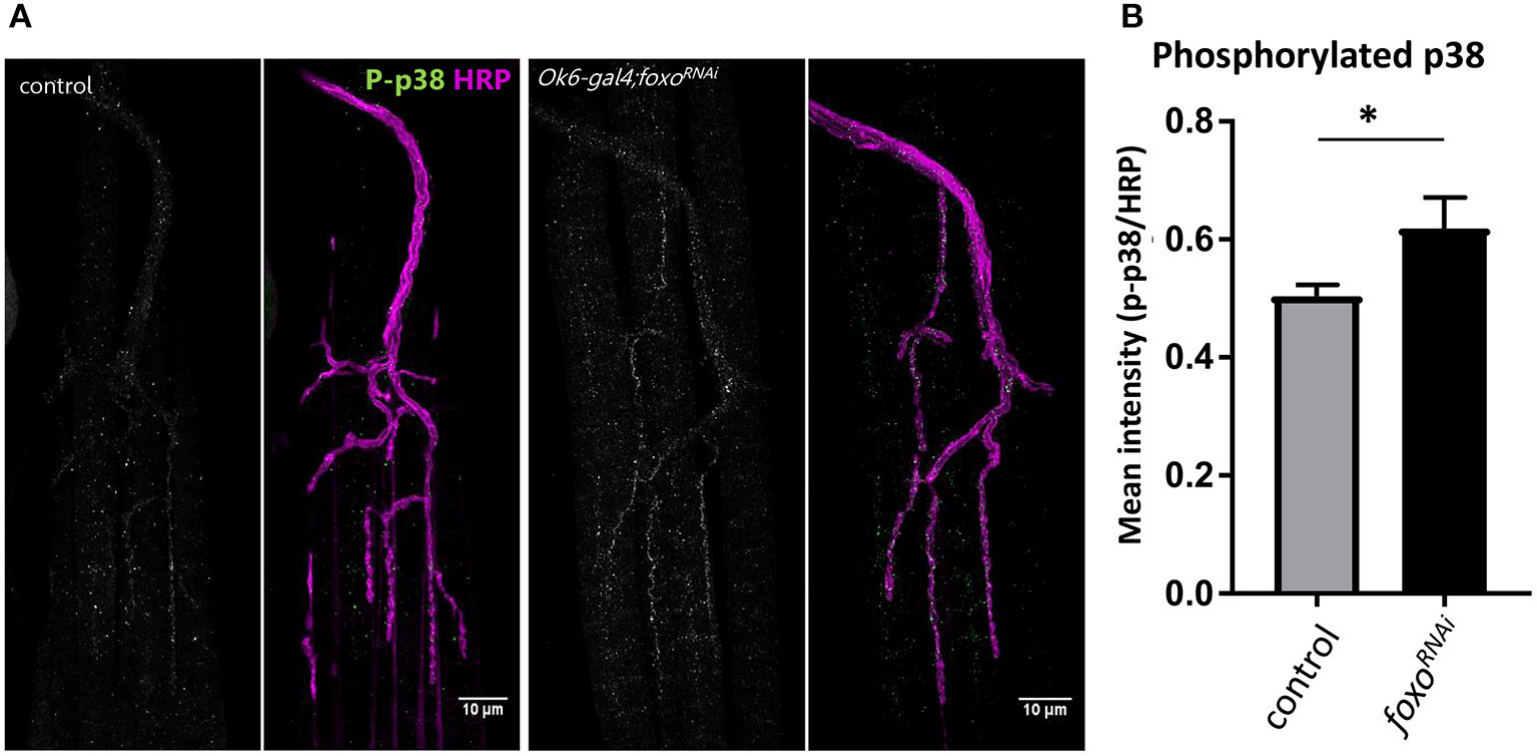
Motor neuron FOXO inhibits MAPK activation. **(A)** Representative images of 1-week old fly NMJs. Colabeled with anti-phosphorylated-p38 and anti-HRP. Scale bar 10 μm. **(B)** Relative intensity of P-p38 at anti-HRP masked areas. Intensity was normalized to anti-HRP intensity. *P < 0.05. Number of NMJs used: ok6-GAL4 >ywR 1wk = 9; ok6-GAL4; foxo-RNAi >ywR 1 wk = 10.

We further confirmed that *foxo* knockdown significantly increased the phosphorylation of p38 in the motor neuron (Figures 5A,B), suggesting that foxo might suppress MAPK signaling in the motor neuron.

## Discussion

Foxo is known to act a regulator of synaptic plasticity and microtubule stability (Howlett et al., 2008; Nechipurenko and Broihier, 2012). Although we have seen foxo's regulation at the NMJ during development, it is unclear the role it plays in NMJ maintenance during aging. Foxo promotes youthful characteristics in many different tissues and has been shown to delay the onset of degenerative properties (Salih and Brunet, 2008; Demontis and Perrimon, 2010). Here we demonstrate that foxo is a regulator of NMJ homeostasis in adult motor neurons. Our data suggests that foxo plays a functional role in maintaining the motor neuron during adulthood and can maintain youthful neuronal phenotypes post-development. We have found that motor neuron expressed foxo contributes to the morphology of synaptic contacts and can regulate cytoskeletal and endocytic processes within neuronal tissue. Our results also suggest that TGF-β and p38/MAPK signaling function downstream of foxo to control these processes. The changes in foxo behavior during aging may induce p38 activation, resulting in disruption of homeostatic mechanisms and promoting cellular senescence.

In the present study, we show that loss of foxo causes morphological changes to synaptic structures in young adult female drosophila. While foxo mutants have been previously shown to have larger boutons compared to controls during development, they were not reported to have any changes in branch length or number, nor were any differences reported in bouton number (Nechipurenko and Broihier, 2012; McLaughlin et al., 2016). We have found this not to be the case in the adult. Our results showed that at 1-week post eclosion, foxo mutant flies had shorter branches compared to control flies. They also had a significantly larger number of branches, which ultimately resulted in a larger bouton number per muscle tissue. We hypothesize that this increase in branching may be due to a compensatory response, as foxo mutants have been shown to have reduced neurotransmission (Howlett et al., 2008; Mahoney et al., 2016), which may cause a need for more synaptic contacts. Additionally, *foxo* mutants are shown to have increased microtubule stability due to reduced microtubule sliding (McLaughlin et al., 2016). This may inhibit growth to postsynaptic targets, resulting shorter branches. The increase in branch number is contrary to what is observed in *Drosophila* larval dendrites, where loss of foxo reduces arborization (Sears and Broihier, 2016). foxo has also been shown to reduce neurite branching in aged *C. elegans* downstream of both JNK and Insulin signaling (Tank et al., 2011). However, increases in ROS can induce overgrowth at the *Drosophila* NMJ, and foxo is known to mitigate oxidative stress through antioxidant transcription activation (Essers et al., 2004; Milton et al., 2011). This increase may also be the result of ineffective pruning during metamorphosis.

Active zone number is proposed to proportionally increase with bouton size under a healthy aging model (Wagner et al., 2015). Although there was no significant difference between average AZ number between controls and foxo mutants, we did observe more AZ's per bouton in the mutants. Previous studies have shown that accumulation of Brp in axons can limit active zone number at synaptic terminals (Barber et al., 2018). Foxo mutant larvae have been shown to have accumulations of Brp in the main axon (McLaughlin et al., 2016), but we were unable to capture this in the adult. Based on our current data, we cannot determine whether foxo influences active zone number. Further experimentation using Electron Microscopy of t-bars in presynaptic area could answer this question.

Aging neurons undergo cytoskeletal changes in order to maintain plasticity and synaptic contact to muscle tissue (Mattson and Magnus, 2006). As previously mentioned, foxo has been shown to regulate the axonal cytoskeleton and neuronal plasticity. During development, foxo mutant larvae have increased futch positive looping and acetylated alpha tubulin is present in the terminal boutons (Nechipurenko and Broihier, 2012). Boutons were also shown to have altered Ac-Tub bundling patterns compared to controls (McLaughlin et al., 2016). In our adult flies, we observed an increase in sinusoidal Ac-Tub morphologies among presynaptic branches, and this is likely a contributing factor to the truncation of established branches during aging. Dynamic stretching of axons leads to undulating distortions and mechanical failure along the axon (Tang-Schomer et al., 2010). These events have been well-studied in injury models, but less so in aging, where regeneration of axonal damage becomes altered (Kleele et al., 2014; Hill et al., 2016; Geoffroy et al., 2017). Additionally, inhibition of dynein has been shown to cause bending in microtubules (Kent et al., 2016). Splayed branching in foxo mutants and motor neuron knockdown flies may be an artifact from the altered bundling pattern observed in the larvae (McLaughlin et al., 2016). While the cause of this is unknown, all microtubule strands appear to be in the same phase of action, in that they are either all straight or all undulating. Additionally, in developing fly larvae, a constitutively active form of foxo caused severe microtubule destabilization, while overexpression of wild-type foxo did not have the same disruption (Nechipurenko and Broihier, 2012). This may indicate that foxo nuclear activity is not solely responsible for maintaining motor neuron integrity.

Endocytosis and exocytosis play important roles in maintaining both the pre- and postsynaptic regions. Endosome-to-lysosome trafficking is disrupted in aging motor neurons, and multivesicular bodies, which are prerequisites for late endosomes, have been shown to accumulate in the presynaptic space during aging (Forester et al., 2010; Wagner et al., 2015). Additionally, disruption of this shuttling is a known symptom of neurodegenerative diseases (Takahashi et al., 2002). In our foxo knockdown flies, we found an increase in the number of late endosomes associated with the axon. This was also observed with aging. Late endosomes are involved for the shuttling of damaged cargo to autophagosomes and lysosomes for degadation, which may indicate an increase in autophagy as a protection mechanism against harmful stress (Maiuri and Kroemer, 2015; Kaur and Lakkaraju, 2018). It has been shown that vesicle axonal transport declines with age, as does lysosome acidification (Forester et al., 2010; Milde et al., 2015; Vagnoni and Bullock, 2018). Although foxo has been shown to promote Rab7 protein expression to promote autophagy in cardiomyocytes, this was in a glucose restricted model and suggests a different mechanism than what we are observing with age (Hariharan et al., 2010). Apoptotic neurons can activate glial innate immunity mechanisms, which involved upregulation of endocytic vesicles in glia to eliminate debris. foxo null-mutants have increased apoptotic debris in the brain during development, and loss of foxo expression in larval cortical glia has reduced phagocytosis and enhanced neurodegeneration (McLaughlin et al., 2019). It is therefore possible that loss of foxo in the motor neuron results in the exportation of damaged cargo to the glia for elimination. However, more experimentation needs to be done to determine the location and underlying causes for this increase in late endosomes. Meanwhile, overexpression of foxo delays late endosome increase in our adult flies.

Through our epistasis analysis, we found knocking down members of MAPK and TGFβ signaling have a profound rescue effect on the late endosome accumulation seen in foxo-RNAi flies. We observed that knockdown of the activin receptor *babo* was able to rescue late endosome accumulation. TGFβ signaling has been shown to regulate axon development and Rho GTPase activity in mushroom bodies and dictates postsynaptic density abundance at the NMJ through motor neuron anterograde signaling (Ng, 2008; Kim and O'Connor, 2014). TGFβ signaling has also been shown to promote aging in cardiac tissue (Chang et al., 2019). We also observed that the MAP kinase p38b was able to rescue late endosome upregulation near the control baseline. In ALS mouse models, inhibition of p38 is able to rescue axonal retrograde trafficking (Gibbs et al., 2018), and p38b is specifically shown to regulate synaptic morphology in drosophila, though in response to stress both p38a and p38b are show to act redundantly in Drosophila, mammals, and yeast (Adachi-Yamada et al., 1999; Klinedinst et al., 2013). Overexpression of MAPK has been shown to increase larval bouton number, and p38 gain of function results in increased oxidative stress, a known target of foxo activity (Koh et al., 2002; Zhan et al., 2015). In mammals, activation of p38/MAPK is associated with increased neuroinflammation, neurodegenerative disease progression, and disruption of synaptic function (Correa and Eales, 2012; Falcicchia et al., 2020). Additionally, TGFβ has been shown to activate p38 independently of SMAD and can induce cell apoptosis through this pathway (Yu et al., 2002). p38 activation has also been associated with increased ROS formation and the promotion of pro-inflammatory cytokines (Zhao et al., 2018). In neuronal tissue, increased ROS and inflammation result in the activation of microglia, with chronic activation being able to induce neuronal damage and apoptosis (Dheen et al., 2007; Gwak et al., 2017). p38 signaling has been shown to regulate Rab7 expression in response to interleukin cytokines (Bhattacharya et al., 2006), further supporting that the increase in Rab7 observed during aging and under foxo knockdown may be a response to increased inflammation.

In our screen we also targeted *rolled* (*rl*), which is the Drosophila ortholog of ERK1/2 (Biggs et al., 1994). We expected to see a decrease in late endosome accumulation but were surprised to find there was no change. We hypothesize that this is due to the potential for two forms of ERK signaling. These two mechanisms of ERK signaling may control opposing processes of synaptic transmission. *Rolled* hypomorphs show a reduced excitatory junction potential, similar to what is seen in foxo hypomorphs, suggesting reduction of *rolled* plays a part in synaptic transmission that may be independent of its role in late endosome shuttling (Wairkar et al., 2009). Additionally, blockage of endosome-lysosome fusion will lead to an increase of activated ERK (Ng and Tang, 2008), suggesting it may still have functional consequences downstream of foxo activity.

In summary, we have found that loss of foxo in the adult *Drosophila* results in morphological changes to synaptic structure that resemble aging morphologies. We also show that motor neuron foxo is required for endocytic homeostasis, and enhanced foxo expression during adulthood can delay aging morphologies. We find evidence to support that foxo may act as a repressor of TGFβ and MAPK signaling, particularly p38 signaling, to maintain youthful characteristics at the NMJ. Identifying the cellular mechanisms regulated by these pathways will provide a greater understanding into how synaptic plasticity is maintained and what homeostatic processes preserve synaptic function during aging.

## Methods

### Fly Stocks and Husbandry

The following stocks were used: *ywR* (Rochele), *ywR;*+*:foxo*^21^, *w*^1118^*, foxoC431* (Gift from N. Liu, Chinese Academy of Sciences, Shanghai, China), *Ok6Gal4* (Gift of E McNeill, Iowa State University), *mhcGal4* (Demontis and Perrimon, 2010, Cell). Flies were collected every 24 h, mated than sorted 2–3 days after eclosion. Flies were placed in vials containing standard CSY food. Fly strains were maintained at 25°C with 12-h light/dark cycle, and 60% humidity. Flies were transferred to fresh food every 3 days. Overexpression and TRiP RNAi lines were obtained from the Bloomington stock center unless otherwise specified: UAS-foxo (BDSC_42221), foxo-RNAi #1 (BDSC_32993), foxo-RNAi #2 (BDSC_32427), ATTP40 (y sc v; attP40 (genotype: y[1] sc[1] v[1]; P{y[+t7.7]=CaryP}attP40)), rl-RNAi (BDSC_34855), khc-73-RNAi (BDSC_36733), lqf-RNAi (BDSC_58130), cpx-RNAi (BDSC_42017), unc-104-RNAi (BDSC_58191), UAS-Ank2GFP (Gift from Ronald Dubreuil, University of Illinois at Chicago), Bsk-RNAi (BDSC_57035), kay-RNAi (BDSC_33379), babo-RNAi (BDSC_25933), p38a-RNAi (BDSC_34744), p38b-RNAi (BDSC_29405), p38c-RNAi (BDSC_64846), grk-RNAi (BDSC_55926), bTub60D-RNAi (BDSC_64856), UAS-Thor (BDSC_9147. Fly lines w^1118^;ok6-Gal4 and BL32993 (foxo-RNAi) were crossed to double balancer yw;Sp/Cyo;TM2e/TM6BTbHue (Rochele). Markers were used to select carriers. After Ok6 was combined with the foxo-RNAi, lines were backcrossed for 5 generations before use. Flies containing Ok6 in a yw background were self-crossed to produce a control strain. Line was backcrossed for 5 generations. A daughterless gene switch GAL4 driver was used to validate foxo knockdown and overexpression.

### Immunofluorescence Staining

Flies were subjected to flynap (Carolina, Burlington, NC) and dissected to expose the ventral abdominal muscles [protocol from Wagner et al. (2015)] in Ca^2+^ free saline [128 mM NaCl, 2 mM KCl, 4 mM MgCl_2_(H_2_O)_6_, 35.5 mM sucrose, 5 mM HEPES, 1 mM EGTA, H_2_O, pH 7.2]. Tissue was then fixed in 4% paraformaldehyde for 20 min at room temperature. Tissue was washed in 1X PBST (0.1% Triton X) and blocked with 5% NGS (normal goat serum) for 1 h at room temperature. Samples we incubated overnight at 4°C with 2 ug primary antibodies in 5% NGS 1X PBST [1X phosphate buffer saline, 0.1% Triton X-100]. Primary antibodies used were as follows: anti-BRP (DSHB nc82), anti-Dlg (DSHB 4F3), anti-Rab7 (DSHB), anti-Rab7 (gift from Nakamura lab), anti-Acetlyated Tubulin (Sigma Aldrich T7451), anti-phospho-p38 MAPK (CST 9211). Tissues we washed in 1XPBST and incubated 1.5 h at room temperature in 1XPBST and anti-HRP-594 or HRP-647 (Jackson ImmunoResearch Laboratories Inc, West Grove PA) at a Dilution of 1:200 with secondary antibodies (1:250) and kept in the dark at room temperature for 2 h. Samples were washed in PBST and mounted in Prolong Diamond (Life technologies). For Fat body analysis, pelts were incubated in slowfade gold with DAPI (life technologies) at 4°C overnight prior to mounting.

### Secondary Antibodies

All secondary antibodies used were from Jackson ImmunoResearch Laboratories Inc and were as follows: 488 donkey anti-mouse, 488 goat anti-rabbit, rabbit anti-HRP-594, donkey anti-HRP-647.

### Imaging Analysis and Quantification

Images were captured using an epifluorescence-equipped BX51WI microscope (Olympus, Waltham, MA, USA) and Olympus FV3000 laser scanning confocal (Olympus, Waltham, MA, USA). CellSens software (Olympus, Waltham, MA, USA) was used for deconvolution of stacks. For aging metrics quantifications, maximum Z projections were generated of whole A3 muscle segment. Bouton size and active zone number were quantified in CellSense through a selected region of interest (ROI) aligned to each bouton. Measurements were performed using the “measure and count” module, providing quantification of bouton and active zone number and area. Branch number and branch length were quantified in Fiji (imageJ, Schindelin et al., 2012). Acetylated alpha-Tubulin quantification was performed via manual counting using the volume module in Fluoview software. Rab7 quantification was performed in CellSense using a circle we generated a circular ROI (25.75 μm^2^) to quantify number of punctae. Z-slices quantified were limited to those containing HRP staining at selected regions to avoid non-neuronal associated punctae. ROIs were checked prior to quantification to ensure muscle associated punctae were removed. Intensities for pp38, pERK, and pJNK were conducted using Fiji (NIH) by measuring the sum fluorescence intensity in ROIs defined by HRP immunoreactivity. HRP fluorescence intensity was measured to ensure no differences between control and mutant NMJs. All images were generated using Fiji.

### Quantitative Real-Time PCR

Three-day post eclosion flies were placed on food containing < 0.1% ethanol and 200 uM Mifepristone (Cayman Chemical) or no other additive for 3 days. Whole bodies of female flies were homogenized in TRIzol (Thermo Fisher). RNA was extracted followi ng company procedure. cDNA was generated using qScript (Quanta) and diluted to a 10 uM working solution. Quantitative PCR was run on purified samples (QuantStudio). Sybrrgreen (Life Technologies, CA, USA) was used for chemical detection. The primers used Enrichment was determined based on the double-delta CT value. *Thor* (sense, 5′ –CCAGGAAGGTTGTCATCTCGG-3′, and antisense 5′ – CTCGTAGATAAGTTTGGTGCCTCC-3′), *Rpl32* (sense 5′-AAGAAGCGCACCAAGCACTTCATC- 3′, and antisense 5′ –TCTGTTGTCGATACCCTTGGGCTT-3′).

### Video Capturing

Stacks of the entire A3 muscle region were acquired with Olympus FV3000 laser scanning confocal in the Fluoview software (Olympus, Waltham, MA, USA). Videos were recorded of 3D renderings using the movie module under the volume setting.

### Venn Diagrams

Venn comparisons were performed using the Bioinformatics and Evolutionary Genomics Venn calculator at http://bioinformatics.psb.ugent.be/webtools/Venn/.

### Statistical Analysis

GraphPad Prism (GraphPad Software) was used for statistical analysis. To compare the mean value of treatment groups vs. that of control, either student *t*-test or one-way ANOVA was performed using Tukey multiple comparison. All genetic screen lines were compared to the generated foxo-RNAi line using a student *t*-test. The metrics during aging were analyzed by two-way ANOVA, including Tukey multiple comparisons test. Before analysis, outliers were identified using Robust regression and Outlier removal (ROUT) method (Q = 1%).

## Data Availability Statement

The raw data supporting the conclusions of this article will be made available by the authors, without undue reservation.

## Author Contributions

AB, MS, MB, KC, and PK: data acquisition. AB, EM, and HB: design, analysis, interpretation, and manuscript revision. AB and HB: conception. AB: manuscript writing. HB: resources and funding acquisition.

## Conflict of Interest

The authors declare that the research was conducted in the absence of any commercial or financial relationships that could be construed as a potential conflict of interest.
