# Supplementary material for: FOXO Regulates Neuromuscular Junction Homeostasis During Drosophila Aging
Source: Front Aging Neurosci. 2021 Jan 27;12:567861. doi: 10.3389/fnagi.2020.567861 (PMC7874159; doi:10.3389/fnagi.2020.567861)
Supplement: Supplementary file 1 [file Data_Sheet_1.pdf]

Supplementary Data

Figure S1. A. Raw counts for bouton area **B.** branch length **C.** active zone number per bouton **D.** Average bouton area and branch length for wild-type (w^{1118}) and $foxo^{C431}$. *P< 0.05, ** P<0.01, ***P>0.001, ****P< 0.0001, n.s. not significant. w^{1118} n=5; $foxo^{C431}$ n=5. **E.** Averaged bouton area and branch length. **F.** Raw bouton area and branch length for wild-type (w^{1118}) and $foxo^{C431}$. *P< 0.05, ** P<0.01, ***P>0.001, ****P< 0.0001, n.s. not significant. w^{1118} n=59; $foxo^{C431}$ n=66. **G. F.** Western analysis of FOXO protein abundance in control and *Foxo*^{21} mutant whole-body tissue.

Figure S2. A. Representative images of anti-Ac-Tub staining for wildtype (yw^R) at 1-week and 25-days of age, and $foxo^{21}$ flies at 1 week of age. **B.** Representative images of anti-Ac-Tub staining for control (Ok6-Gal4> yw^R) at 1 week and 25 days of age, and Ok6-Gal4> foxo-RNAi flies at 1 week post eclosion. **C.** Quantification of acetylated alpha-tubulin structure. Significance values are compared to young control for each grouping . *P< 0.05, ** P<0.01, ***P>0.001.

Figure S3. A. Rab7 quantification at 1 week for ok6-Gal4>ywR, ok6-Gal4>foxo-RNAi #1, ok6-Gal4> foxo-RNAi #2. **B.** Thor relative expression for whole body tissue. Daughterless-Gal4 activated with 200mM of RU486. **C.** Quantification of FOXO protein intensity in fat body nuclei after RU486 induced knockdown. * P< 0.05. **D.** Representative images of fat body tissue for control (S106-GS-GAL4> w^{1118}) and FOXO RNAi (S106-GS-Gal4>BL32993). S106-Gal4 activated with 200mM of Mifepristone (RU). Staining with anti-FOXO and DAPI. Scale Bar 10µm**,** n=5**.**

Figure S4. A. Rab7 relative intensity quantification at 1 week for ok6-Gal4>ywR, ok6-Gal4>foxo-RNAi. **B.** Rab7 intensity quantification Rab7 quantification at 2-days for ok6-Gal4>ywR, ok6-Gal4>foxo-RNAi **C.** Bouton area quantification at 2-days for ok6-Gal4>ywR, ok6-Gal4>foxo-RNAi **D.** Ac-Alpha tubulin quantification at 2-days for ok6-Gal4>ywR, ok6-Gal4>foxo-RNAi **E. .** Quantification of Rab7 for epistasis flies. Values are set as a percentage with ok6-Gal4>Attp40 as baseline. *P< 0.05, n.s. – not significant.

Figure S5. Representative confocal images from genetic screening. A. anti-rab7 and anti-HRP immunofluorescence for ok6;$foxo^{RNAi}$> $p38b^{RNAi}$, ok6;$foxo^{RNAi}$> rl^{RNAi}, ok6;$foxo^{RNAi}$> $p38a^{RNAi}$. B. anti-rab7 and anti-HRP immunofluorescence for control (ok6-gal4> yw^R) ; ok6;$foxo^{RNAi}$> yw^R ; ok6;$foxo^{RNAi}$> $babo^{RNAi}$, **C.** anti-Ac-Tub and anti-HRP immunofluorescence for ok6;$foxo^{RNAi}$> $p38b^{RNAi}$, ok6;$foxo^{RNAi}$> $babo^{RNAi}$, ok6;$foxo^{RNAi}$> yw^R.

Figure S1

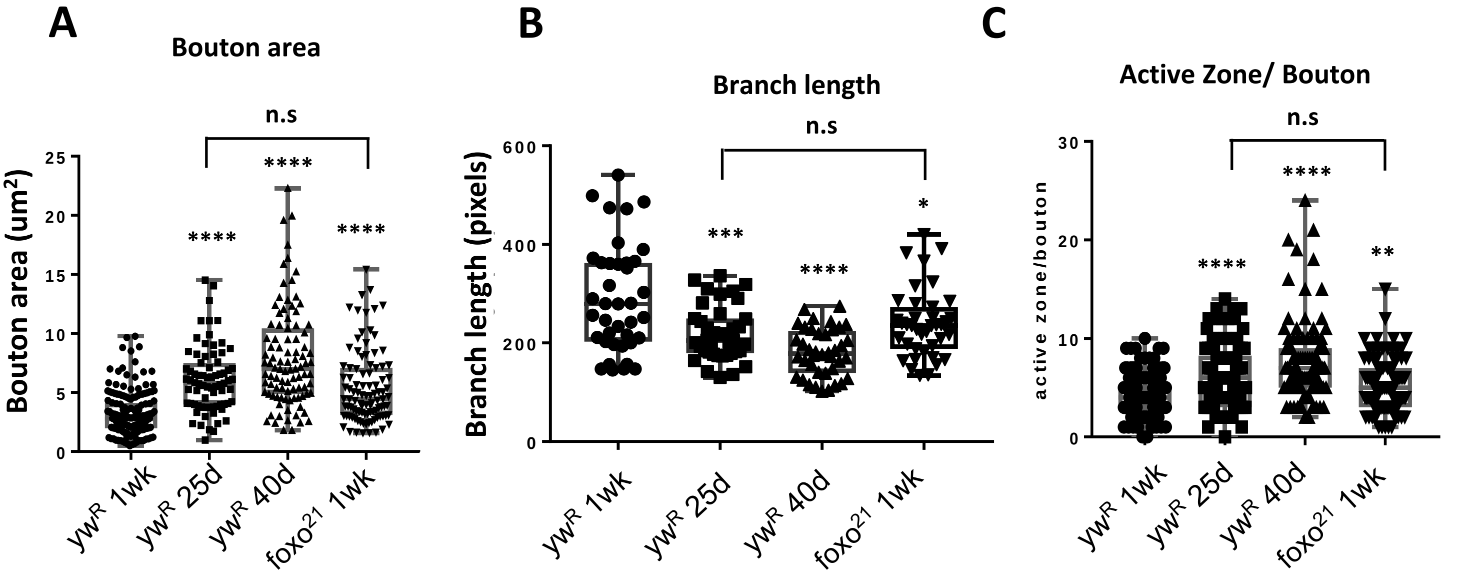

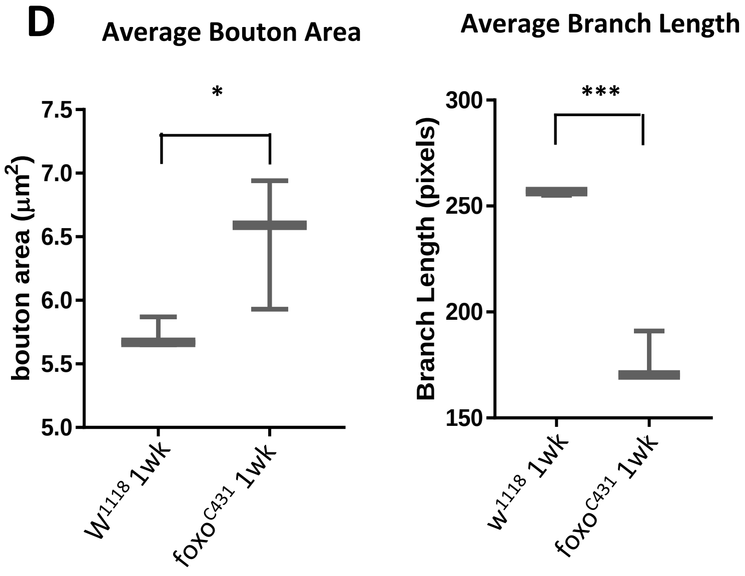

E

	Size of boutons (um²)	Branch length (pixels)
w^{1118} 1wk	5.53 ± 2.47	256.4 ± 49.85
$foxo^{C431}$ 1wk	6.59 ± 2.94	175.2 ± 55.32

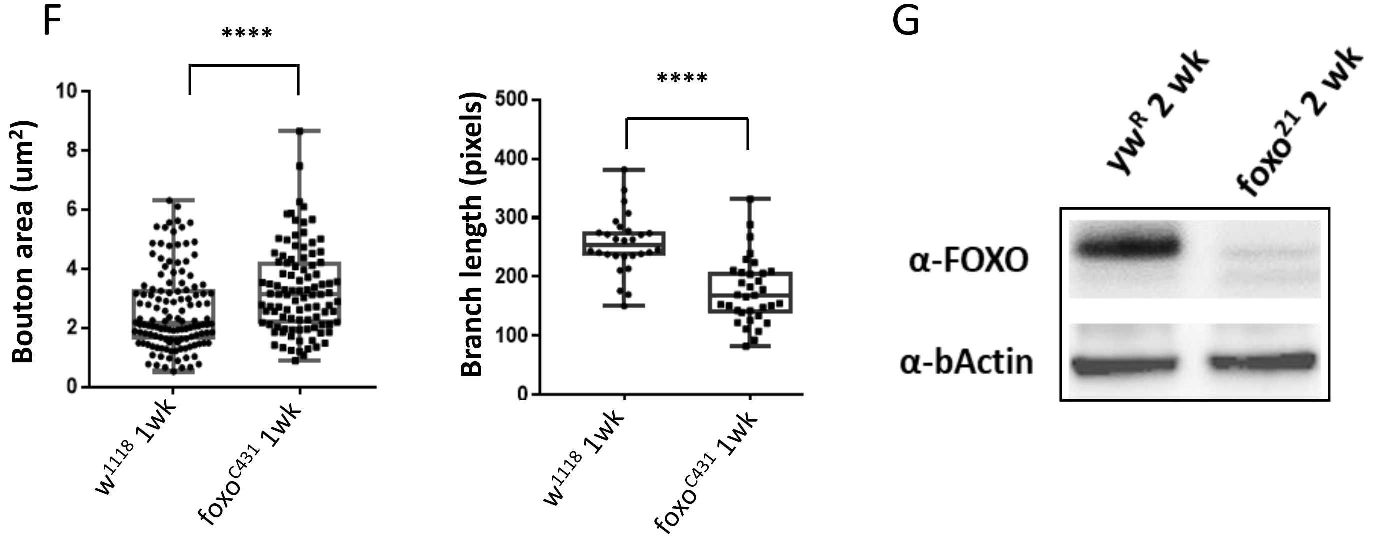

Figure S2

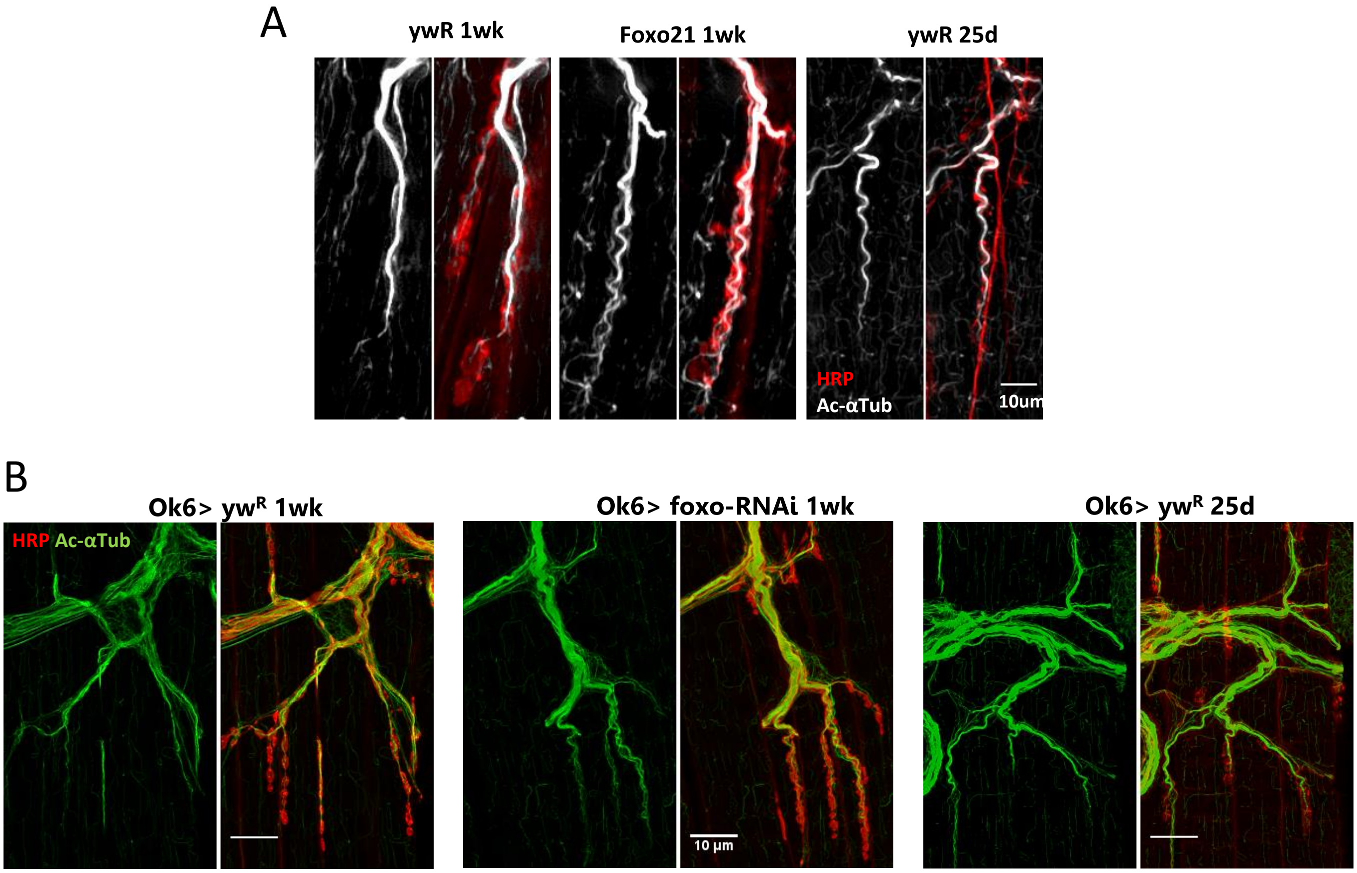

C

	Straight	Undulating
ywR 1wk	69.24%	30.76%
yw^R 25d	40.38%*	59.62%*
yw^R 40d	30.00%**	70.00%**
$foxo^{21}$ 1wk	46.83%*	53.17%*
ok6 > control 1wk	67%	33%
ok6 > foxo-RNAi 1wk	34.27%***	65.73%***
ok6 > control 25d	38.64%**	61.36%**

Figure S3

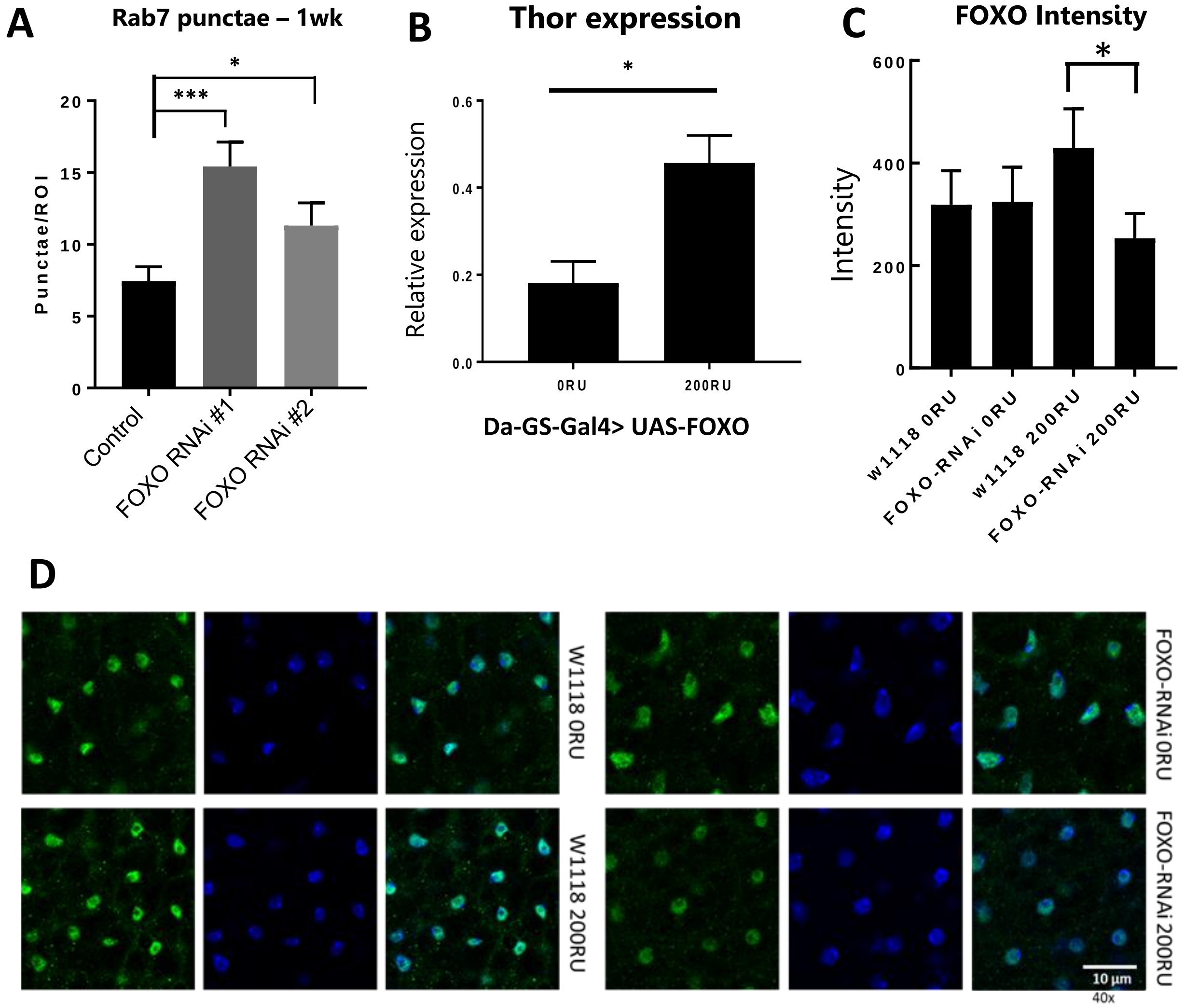
A
Rab7 punctae – 1wk
Punctae/ROI
***
*
Control
FOXO RNAi #1
FOXO RNAi #2
B
Thor expression
Relative expression
*
0RU
200RU
Da-GS-Gal4> UAS-FOXO
C
FOXO Intensity
Intensity
*
w1118 0RU
FOXO-RNAi 0RU
w1118 200RU
FOXO-RNAi 200RU
D
W1118 0RU
W1118 200RU
FOXO-RNAi 0RU
FOXO-RNAi 200RU
10 µm
40x

Figure S4

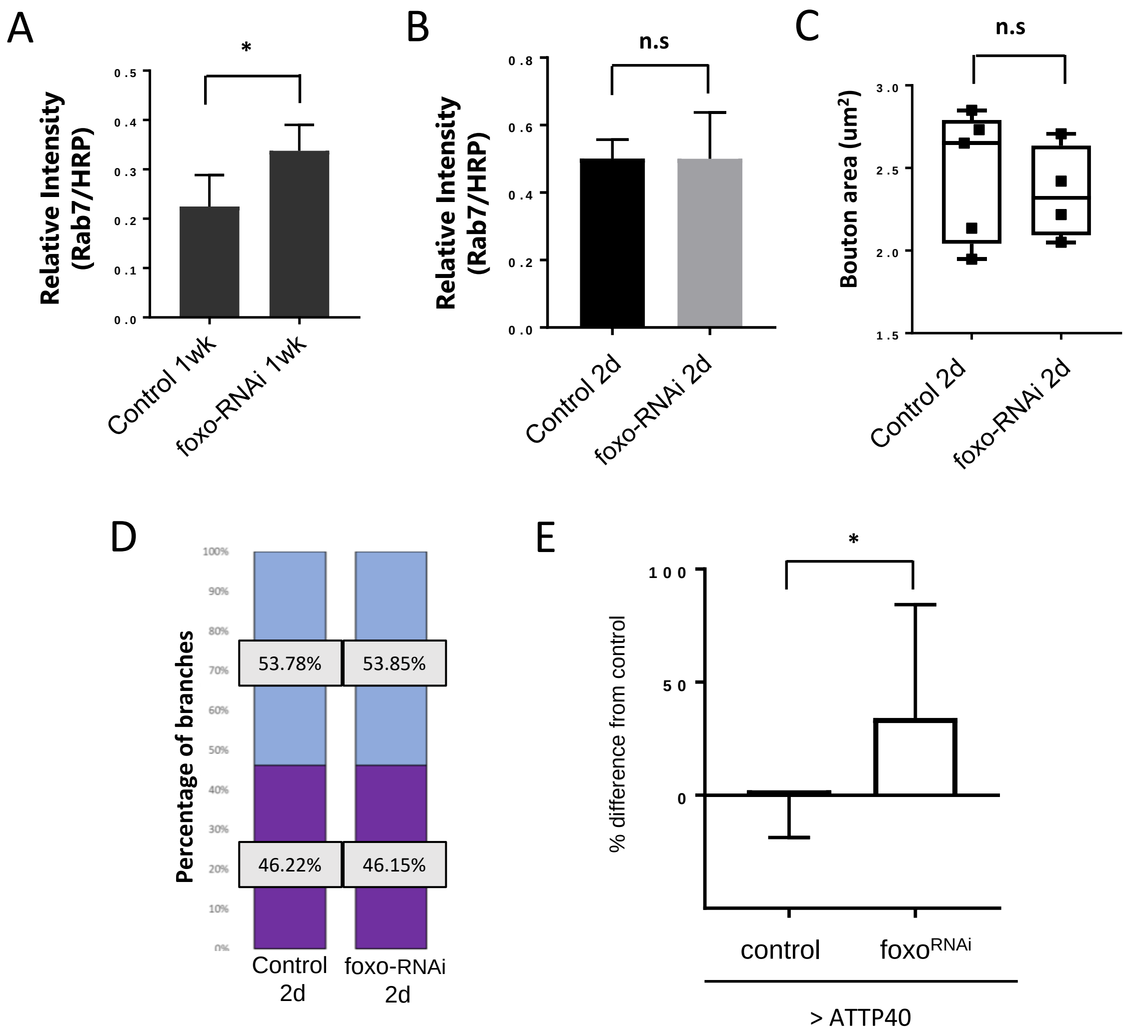

A
*
Relative Intensity (Rab7/HRP)
0.5
0.4
0.3
0.2
0.1
0.0
Control 1wk
foxo-RNAi 1wk
B
n.s
Relative Intensity (Rab7/HRP)
0.8
0.6
0.4
0.2
0.0
Control 2d
foxo-RNAi 2d
C
n.s
Bouton area (um2)
3.0
2.5
2.0
1.5
Control 2d
foxo-RNAi 2d
D
Percentage of branches
53.78%
53.85%
46.22%
46.15%
Control 2d
foxo-RNAi 2d
E
*
% difference from control
100
50
0
control
foxoRNAi
> ATTP40

Figure S5

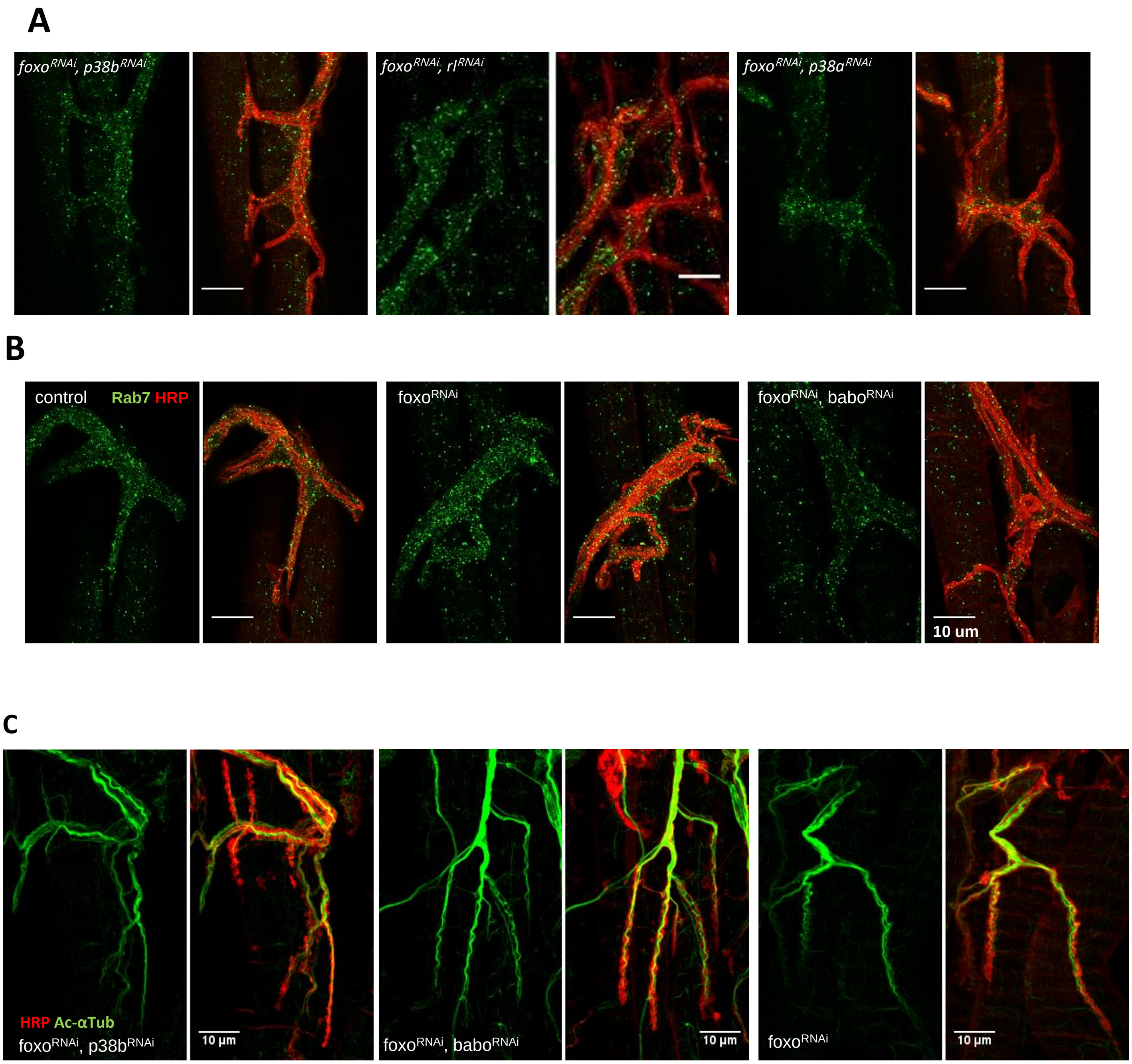
A
foxo^{RNAi}, p38b^{RNAi}
foxo^{RNAi}, rl^{RNAi}
foxo^{RNAi}, p38a^{RNAi}
B
control
Rab7 HRP
foxo^{RNAi}
foxo^{RNAi}, babo^{RNAi}
10 um
C
HRP Ac-αTub
foxo^{RNAi}, p38b^{RNAi}
10 μm
foxo^{RNAi}, babo^{RNAi}
10 μm
foxo^{RNAi}
10 μm
